# Recurrent c.‐11C>T change located upstream of the normal ATG initiation codon of *ANKH* causes self‐limited familial infantile epilepsy

**DOI:** 10.1111/epi.18504

**Published:** 2025-06-27

**Authors:** Josua Kegele, Hendrik Juenger, Harald Frantzmann, Dieter Gläser, Marc Sturm, Holger Lerche, Tobias B. Haack, Ingrid Bader

**Affiliations:** ^1^ Department of Neurology and Epileptology, Hertie Institute for Clinical Brain Research University of Tübingen Tübingen Germany; ^2^ EpiCARE European Reference Network; ^3^ Klinik für Kinderheilkunde, Jugendmedizin, und Neonatologie Klinikum Kempten Kempten Germany; ^4^ Kinder‐ und Jugendarztpraxis Memmingen Germany; ^5^ MVZ Genetikum Neu‐Ulm Germany; ^6^ Institute of Medical Genetics and Applied Genomics University of Tübingen Tübingen Germany; ^7^ Center for Rare Diseases University of Tübingen Tübingen Germany; ^8^ Genomics for Health in Africa Africa–Europe Cluster of Research Excellence

**Keywords:** *ANKH*, chondrocalcinosis, epilepsy, febrile seizures, self‐limited familial infantile epilepsy

## Abstract

**Objective:**

Pathogenic *ANKH* variants are a known cause of chondrocalcinosis (Online Mendelian Inheritance in Man [OMIM] #118600) and craniometaphyseal dysplasia (OMIM #123000). Here, we describe the phenotype and genotype of autosomal dominant infantile epilepsy caused by a c.‐11C>T change upstream of the gene's normal ATG initiation codon of *ANKH* in a family of southern Italian descent; we correlate the phenotype with known epilepsy syndromes and provide the first evidence of recurrence of this particular *ANKH* variant.

**Methods:**

Phenotyping and genotyping (short‐read exome/genome sequencing) was performed on six members of a family with self‐limited familial infantile epilepsy (SeLFIE).

**Results:**

We describe a family with six individuals who presented with infantile onset epilepsy. All affected family members experienced focal and/or bilateral tonic–clonic seizures, sometimes triggered by fever or infection, with seizure onset predominantly before the age of 2 years. Patients responded well to antiseizure medication, and seizures resolved completely before the age of 4 years. Short‐read genome/exome sequencing and comparative bioinformatic analysis of the variants of five affected individuals and one unaffected individual revealed *ANKH* c.‐11C>T as the causative pathogenic variant in this family, segregating with the disease.

**Significance:**

To our knowledge, we report the second family with autosomal dominant epilepsy caused by an *ANKH* c.‐11C>T variant. The pediatric phenotype closely resembles that of the previously reported British family, suggesting low phenotypic heterogeneity, and aligns with SeLFIE. *ANKH‐*associated epilepsy should be considered in SeLFIE, especially in cases with a family history of chondrocalcinosis or recurrent acute joint pain episodes.

**Key points**

- Here, we report the second family with autosomal dominant self‐limited infantile onset epilepsy associated with the *ANKH* c.‐11C>T variant.
- Our work provides evidence for the recurrence of the *ANKH* variant c.‐11C>T and that this particular variant is causal for epilepsy in humans.
- The phenotype observed in affected individuals can be classified as SeLFIE.

## 1 INTRODUCTION

The human *ANKH*‐gene is located on the short arm of chromosome 5 in 5p15.2. Pathogenic variants in *ANKH* (Online Mendelian Inheritance in Man [OMIM] #605145) have been associated with craniometaphyseal dysplasia^1^ (OMIM #123000) and autosomal dominant inherited chondrocalcinosis 2 (CCAL2; OMIM #118600) in humans.^2^ CCAL2 is characterized by the deposition of calcium pyrophosphate crystals in the joints and manifests with episodes of acute arthritis or chronic arthropathy with age at onset typically in the third decade. The original publication, which for the first time described human *ANKH* variants that were associated with autosomal dominant chondrocalcinosis (CC), reported three families carrying three different *ANKH* variants (‐11C>T, M48T, and E490del). A UK family, in which a c.‐11C>T change created a novel ATG initiation codon in the 5′ untranslated region (UTR) of *ANKH*, was unique in that adult onset CC was preceded by repeated self‐limiting seizures during childhood. The N‐terminal extra four amino acids extension found in the UK family was hypothesized to produce a new activity or interaction in neural cells, a known site of ANK expression in mice and humans.^2^, ^3^ In all 13 affected members of the UK family, the c.‐11C>T variant segregated with adult onset CC preceded by febrile seizures and/or early onset and self‐limiting epilepsy. To our knowledge, no other individuals with *ANKH*‐associated autosomal dominant epilepsy have been reported in literature since then.

Here, we describe the second family with *ANKH*‐related self‐limited autosomal dominant infantile epilepsy.

## 2 MATERIALS AND METHODS

Written informed consent was obtained from individuals II.1, II2., III.1, III.2, II.3, and III.4 to participate in the study and to publish clinical and genetic information. The study was conducted according to local regulations and was approved by the ethics committees involved (ethics committee of the Faculty of Medicine at University Hospital Tübingen, Germany, project number 198/2010BO1). All study‐related procedures were performed in accordance with the ethical standards outlined in the Declaration of Helsinki and its later amendments.

A comprehensive personal clinical assessment was conducted on three affected individuals (III.1, III.2, and II.2). Clinical data concerning individuals I.2, II.3, and III.4 were obtained through interviews with family members II.1 and II.2.

Clinical data were collected by the investigators directly from the patient, from medical charts, or from the physician in charge. For genetic testing, EDTA‐blood samples were taken and DNA was extracted using standard procedures.

Regarding genetic testing, for patients II.1, II.2, and III.1, trio‐exome sequencing was performed from genomic DNA extracted from peripheral blood. Coding regions and adjacent intronic regions were enriched using a SureSelect XT Human All Exon kit v6 (Agilent Technologies) for subsequent sequencing as 2 × 100‐bp paired‐end reads on a NovaSeq6000 system (Illumina).

Additional genome sequencing was performed on genomic DNA extracted from peripheral blood for patients II.3, III.2, and III.4. Genomic DNA was processed with the TruSeq PCR‐Free Library Prep Kit (Illumina) and subsequently sequenced as 2 × 150‐bp paired‐end reads on a NovaSeq6000 system (Illumina).

Generated sequences were analyzed using the megSAP pipeline (https://github.com/imgag/megSAP). Clinical variant prioritization included several filtering steps including a search for rare (minor allele frequency < .1% in gnomAD and an in‐house database) variants in genes that have been associated with the patient's phenotype according to an in‐house standard operating procedure. Clinical variant prioritization was conducted independently by two trained diagnostic molecular geneticists.

## 3 RESULTS

### 3.1 Clinical data

#### 3.1.1 Patient III.2

Delivery of III.2 was premature by emergency cesarean section due to placental abruption. Birth weight of the female was 1800 g, and neonatal intensive care/continuous positive airway pressure treatment was necessary. After 10 days, the neonate was discharged with a body weight of 2000 g. Development was normal and uneventful except for a respiratory syncytial virus infection at 3 months of age. At age 10 months, she was admitted to the hospital because of a first unprovoked seizure. Another generalized tonic–clonic seizure was observed upon admission, prompting the initiation of phenobarbital treatment (11.6 mg/kg). One year later, an attempt was made to gradually discontinue phenobarbital, but shortly thereafter, she experienced a series of seizures, comprising atonic and generalized tonic–clonic seizures, necessitating the initiation of a maintenance therapy with valproate (300 mg/day, 25 mg/kg, serum level = 54 mg/L).

At age 30 months, she experienced recurrent focal nonaware motor seizures, particularly tonic seizures of the right arm, provoked by a rotavirus infection. Cerebral magnetic resonance imaging (MRI) at 33 months of age showed accentuation of the inner and outer liquor systems and minimal paraventricular gliosis, with no epileptogenic lesion detected. Repetitive electroencephalography (EEG) yielded normal results. Antiseizure medication was stopped at the age of 5 years, and she remained seizure‐free.

**FIGURE 1:**
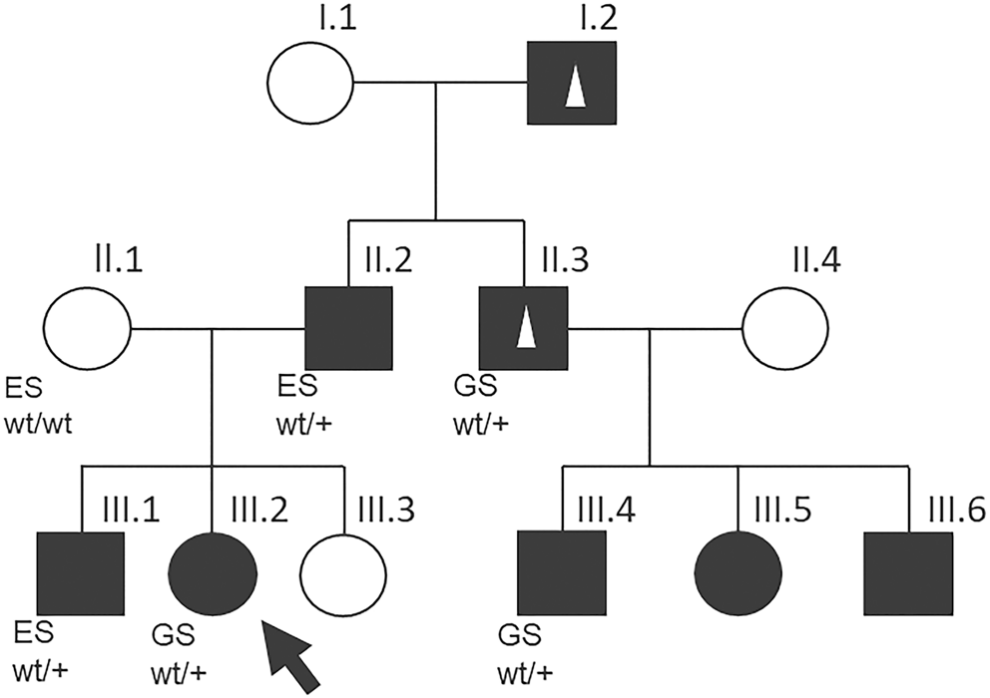
Pedigree of the affected family carrying the variant *ANKH* c.‐11C>T. Squares = males; circles = females; white squares/circles = unaffected; black squares/circles = affected by infantile seizures; small white triangle = affected by joint pain/chondrocalcinosis in adulthood; black arrow = index patient (III.2). ES, exome; GS, short‐read genome sequencing performed in this patient; wt, wild‐type allele; +, allele carrying the variant *ANKH* c.‐11>T.

At the last follow‐up, at 7 years of age, she was still seizure‐free without medication but was receiving logotherapy and ergotherapy. In terms of cognition, according to the mother, she demonstrated superior performance compared to her affected brother (Figure 1; III.1) but lagged behind her unaffected younger sister (Figure 1; III.3). She commenced schooling at 6 years 11 months of age. Neurological examination revealed discrete fine motor and coordinative deficits but was otherwise unremarkable. The EEG showed an 8/s alpha rhythm with normal background activity and no epileptiform discharges. Her growth parameters were normal at the last follow‐up at the age of 5 years (Table 1). The family history was positive for epilepsy, indicating an autosomal dominant inheritance pattern (Figure 1). The clinical information for all affected individuals in the family is summarized in Table 1.

**TABLE 1:** Overview of clinical data from tested family members.

	Index (III.2)	Brother (III.1)	Father (II.2)	Grandfather (I.2)	Uncle (II.3)	Cousin (III.4)
ANKH (ENST00000284268.8)	c.‐11C>T	c.‐11C>T	c.‐11C>T	No genetic testing	c.‐11C>T	c.‐11C>T
Genomic coordinate (hg38)	chr5:14871458G>A	chr5:14871458G>A	chr5:14871458G>A	chr5:14871458G>A	chr5:14871458G>A	chr5:14871458G>A
Zygosity	Heterozygous	Heterozygous	Heterozygous	No genetic testing	Heterozygous	Heterozygous
Gender	Female	Male	Male	Male	Male	Male
Age	7 years	8 years	35 years	68 years	39 years	12 years
Pregnancy, delivery	Placenta abruption; cesarean section at 32 + 1 week of gestation	Stalled birth, cesarean section at 40 + 2 week of gestation	Normal at 39th week of gestation	–	–	–
Length/weight/OFC at birth (centile)	42 cm (9th–25th)/1803 g (50th−75th)/28 cm (9th−25th)	3530 g (85th)/54 cm (35th)/33 cm (3rd)	53 cm (91st–98th)/3910 g (91st)/35 cm (50th–75th)			
Height/weight/head circumference at age of last examination (centile)	5 years of age (18th/31st/56th)	–	–	–	–	–
Epilepsy type	Focal genetic epilepsy	Focal genetic epilepsy	Unclassified	Unclassified	Unclassified	Unclassified
Seizure type	Focal tonic nonaware seizures, also cluster of seizures	Focal to BTCS	Unclassified	Unclassified	Febrile seizure only	Unclassified
Age at investigation	7 years	8 years	35 years	–	–	–
Age at onset of seizures	10 months	13 months	1 year	<5 years	<1 year	–
Age at last seizure	3 years (provoked)	3 years (provoked)	1 year	<5 years	<1 year	–
Seizure outcome	Seizure‐free without medication	Seizure‐free without medication	Seizure‐free without medication	Seizure‐free without medication	Seizure‐free without medication	–
Trigger	Infection	Fever, afebrile infection	None	None	Fever	–
Development	Normal milestones	Normal milestones	Normal	–	Normal	–
Antiseizure medications	PB, VPA	PB, LEV, BRV, good response	PRM	None	PRM	–
Duration of antiseizure medication treatment	12 months (10–22 months of age) and 4 years (22 months of age until 6 years of age)	2 years (1–3 years of age) and 3 years after encephalitis	2 years	None	“Several years”	–
Comorbidities	Hemangioma, parieto‐occipital right (extracranial); rotavirus gastroenteritis at age of 3 years triggering seizures; transient ataxia	Encephalitis at age 3; postencephalitis: mild delayed motor and speech development, not fully recovered	None	Rheumatoid disease with joint pain and common use of ibuprofen, no exact diagnosis available, age at onset not known	Chronic pain in the knees, starting at the age of 30 years, taking painkillers regularly, also suffers from joint pain attacks in the knees	–
Neurological examination	Mild fine motor deficit and coordination deficit	Mild fine motor deficit, logopedic therapy, bad school performance	Normal	–	–	–
EEG	Normal	Normal	–	–	–	–
cMRI	Accentuation of the inner and outer liquor systems and minimal paraventricular gliosis, no epileptogenic lesion	Normal (during encephalitis T2 hyperintensities in the trigonum collaterale and the splenium, follow‐up MRI 4 years later: normal)	–	–	–	–

Abbreviations: BRV, brivaracetam; BTCS, bilateral tonic–clonic seizures; cMRI, cerebral magnetic resonance imaging; EEG, electroencephalography; LEV, levetiracetam; MRI, magnetic resonance imaging; OFC, occipitofrontal head circumference; PB, phenobarbital; PRM, primidone; VPA, valproic acid.

### 3.2 Sequence analysis, variant interpretation, databases, and cosegregation analyses

**FIGURE 2:**
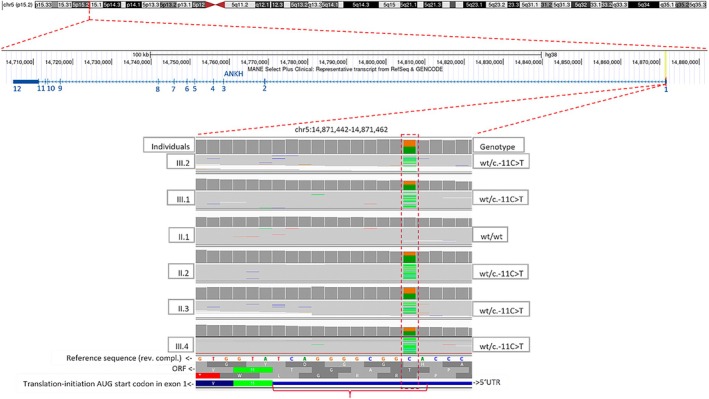
Top: Sketch of chromosome 5. Middle: Zoom‐in to 5p15.2 of the reference‐sequence (hg38) showing the genomic organization of all 12 exons of the MANE transcript (ENST00000284268.8) of *ANKH* as annotated in the UCSC Genome Browser. Bottom: Zoom‐in to exon 1 of *ANKH* and screenshot from the Integrative Genomics Viewer showing the reads at the position with the heterozygous variant *ANKH* c.‐11C>T (dashed rectangle) in the 5′ untranslated region (UTR) from six individuals of the family. The C>T change leads to an alternative ATG (AUG) start‐codon, elongating (bracket) the N‐terminal open reading frame (ORF) for four additional residues (Met‐Ala‐Gly‐Thr). The mother (II.1) does not carry the variant. rev. compl, reverse complementary; wt, wild‐type.

Genetic analysis revealed the heterozygous variant *ANKH* (ENST00000284268.8):c.[‐11C>T];[=] p.[?];[=] in all affected tested individuals, which creates an alternative start‐codon in the 5′UTR of exon 1 (see Figure 2). The variant is not present in the genomes of a control database in 151 942 alleles (gnomAD v4.1.0) but cosegregates with the disease in all five tested individuals of our family (Figures 1 and 2).

Currently, 19 different pathogenic *ANKH* variants are listed in the Human Gene Mutation Database (HGMD Professional 2024.4, March 12, 2025): 11 missense variants, one splice variant, five small in‐frame deletions, one small indel, and one regulatory variant (which is the c.‐11C>T change). All of the listed variants are associated with either craniometaphyseal dysplasia (OMIM #123000) or CCAL2 (OMIM #118600). This is also true for the current ClinVar entry concerning the c.‐11C>T change (variation ID: 5196). We resubmitted the variant in ClinVar (submission ID: SUB15160455) with the observed seizure phenotype (Orpha number: 306).

## 4 DISCUSSION

Here, we report the second family with autosomal dominant self‐limited infantile onset epilepsy associated with *ANKH* c.‐11C>T. The epilepsy‐related phenotype can be summarized as epilepsy with focal and/or bilateral tonic–clonic seizures with seizure onset predominantly before 2 years of age. Seizures are sometimes triggered by infection and/or fever. Patients respond well to antiseizure medication, and seizures resolve completely by 5 years of age. The phenotype observed in this family resembles that of a previously reported British family with 13 affected members,^4^ suggesting low phenotypic heterogeneity. In the British family, seizure onset occurred within a similar range (between 6 months and 2 years); the offset of seizure has even been described as at the age of 4 years at the latest in all affected individuals. Fever‐associated seizures were also documented. Seizure clusters have been observed in both families (the UK family and the family of Italian descent described here) as well as encephalitis with subsequent learning disability in one member of each family. Mild learning disability has been documented in three affected individuals of the British family, which is in line with the neurocognitive abnormalities diagnosed in our patients III.1 and III.2.

The phenotype of the affected members of our family shows close similarity with self‐limited familial infantile epilepsy (SeLFIE; Orpha number: 306), formerly known as benign familial infantile epilepsy (OMIM #607745); age at onset (6 months to 2 years), seizure types (focal seizures and focal to bilateral tonic–clonic seizures), occurrence of seizure clusters, good treatment response, normal EEG and MRI, and autosomal dominant inheritance of *ANKH‐*associated epilepsy are in line with SeLFIE,^5^ thus the affected members of our family members may be diagnosed as SeLFIE. We suggest testing patients who are negative for common disease‐causing variants in SeLFIE (*SCN2A, SCN8A, KCNQ2, PRRT2*) for the *ANKH* c.‐11C>T change, especially if there is evidence for a familial arthropathy.

All individuals who were affected by seizures in the British family also suffered from calcium pyrophosphate deposition, suggesting a high penetrance for the arthropathy with onset in adulthood. In our family, two individuals have been reported to experience chronic (I.2) or episodic (II.3) joint pain (see also Table 1). Although an exact diagnosis was not available, the reported symptoms are consistent with *ANKH*‐associated CCAL2.

In vitro analyses have shown that the c.‐11C>T change creates an elongated polypeptide with four additional residues added to the N‐terminus (Met‐Ala‐Gly‐Thr) compared to the wild‐type sequence.^2^ Functional studies demonstrated increased activity (gain of function) of the encoded protein ANKH inorganic pyrophosphate transport regulator, thus increasing intracellular pyrophosphate.^2^ The pathophysiological mechanism that leads to epilepsy is not well understood and has been poorly investigated; upregulation of *ANK*, the rodent homolog of *ANKH*, has been observed following seizure induction in rats, indicating a possible role in neuronal excitation.^6^ It is hypothesized that alterations in intra‐ and extracellular pyrophosphate levels may influence neuronal membrane excitability, contributing to a predisposition to seizures.^4^ Probenecid, an anion transport inhibitor, has been proposed as a potential agent for precision therapy, as it also inhibits ANKH transport activity.^3^ However, it remains unclear whether the altered function of the mutated *ANKH* directly causes epilepsy or interacts with other, yet unidentified pathways involved in epilepsy or neurodevelopment.

Further evidence for a role of ANKH in the central nervous system comes from a consanguineous family where homozygosity of the *ANKH* missense variant (L244S) segregated with an autosomal recessive disorder comprising mental retardation, deafness, ankylosis, and mild hypophosphatemia.^7^

*ANKH*‐associated epilepsy may be underdiagnosed for two reasons; first, the C>T change is located in the 5′UTR region of the gene, 11 nucleotides upstream of the “normal” translation initiation start‐codon, and second because of the currently missing connection of the *ANKH* gene entry with a seizure phenotype in relevant databases that are integrated in standard bioinformatic pipelines for variant calling and variant interpretation (e.g., databases like OMIM and ClinVar). The identification of more epilepsy patients with this variant could potentially lead to the development of therapeutic targets aiming at the prevention not only of epilepsy but also of neurocognitive disabilities and arthropathy.

The study has several limitations. The clinical history of the affected second branch (II.3 and III.4) of the family and of the grandfather (I.2) is limited and may be affected by recall bias. Finally, the proposed core phenotype discussed here is based on data from only two families. Additional reports and long‐term follow‐up of carriers of the *ANKH* c.‐11C>T variant are necessary to further expand the phenotypic spectrum.

## 5 CONCLUSIONS

Our work provides evidence for the recurrence of the *ANKH* variant ‐11C>T. We provide cosegregation data and genotype–phenotype correlation that confirm that this particular variant and the related pathomechanism are causal for autosomal dominant epilepsy in humans. Our study highlights the role of *ANKH* in epilepsy and will help to correctly diagnose infantile epilepsy more often, by shedding new light on this potentially underdiagnosed genetic form of epilepsy.

## AUTHOR CONTRIBUTIONS

**Josua Kegele:** Conceptualization (lead); writing—original draft (lead); formal analysis (equal); writing—review and editing (equal). **Hendrik Juenger**, **Harald Frantzmann**, and **Dieter Gläser:** Writing—review and editing (equal). **Marc Sturm:** Software (lead); writing—review and editing (equal). **Holger Lerche:** Writing—review and editing (equal); supervision (equal). **Tobias B. Haack:** Writing—review and editing (equal); supervision (equal). **Ingrid Bader:** Conceptualization (supporting); writing—original draft (supporting); formal analysis (lead); writing—review and editing (equal).

## FUNDING INFORMATION

J.K. received funding from the University of Tübingen (Fortuene‐Antrag Nr. 301100).

## CONFLICT OF INTEREST STATEMENT

None of the authors has any conflict of interest to disclose. We confirm that we have read the Journal's position on issues involved in ethical publication and affirm that this report is consistent with those guidelines.

## Data Availability

The data that support the findings of this study are available from the corresponding author upon reasonable request.
